# Pandemic (H1N1) 2009 in Captive Cheetah

**DOI:** 10.3201/eid1802.111245

**Published:** 2012-02

**Authors:** Beate Crossley, Sharon Hietala, Tania Hunt, Glenn Benjamin, Marie Martinez, Daniel Darnell, Adam Rubrum, Richard Webby

**Affiliations:** California Animal Health and Food Safety Laboratory, University of California, Davis, California, USA (B. Crossley, S. Hietala);; Safari West, Santa Rosa, California, USA (T. Hunt, G. Benjamin, M. Martinez);; St. Jude Children’s Research Hospital, Memphis, Tennessee, USA (D. Darnell, A. Rubrum, R. Webby)

**Keywords:** pandemics, zoonoses, Acinonyx jubatus, cheetahs, influenza A virus, H1N1 subtype, pandemic (H1N1) 2009, virus, influenza, China

## Abstract

We describe virus isolation, full genome sequence analysis, and clinical pathology in ferrets experimentally inoculated with pandemic (H1N1) 2009 virus recovered from a clinically ill captive cheetah that had minimal human contact. Evidence of reverse zoonotic transmission by fomites underscores the substantial animal and human health implications of this virus.

## Case Report

In November 2009, during the surge of pandemic (H1N1) 2009 cases among humans and ≈7 months after A/California/04/2009(H1N1) was first reported in a child in southern California (*1*), nasal swab specimens from a cheetah (*Acinonyx jubatus*) were submitted to the California Animal Health and Food Safety Laboratory. The 8-year-old cheetah, referred to as animal D, belonged to a privately operated wild animal park in northern California. The animal park veterinarian collected the specimens from animal D for diagnostic testing after severe respiratory symptoms, ptyalism, anorexia, and lethargy were observed in the animal and 3 other cheetahs housed in the same area. The 4 animals had been showing clinical signs of an influenza-like illness the previous week and were being treated with a combination of amoxicillin, enrofloxacin, famotidine, and omeprazole.

At the recommendation of a volunteer worker who was professionally affiliated with the California Public Health Department, a nasal swab sample was obtained from animal D 2–3 days following the onset of clinical signs. The California Public Health Department supplied a sampling kit consisting of cotton-tipped swabs, viral transport media, and a shipping container. A swab sample also was obtained from animal C, another 8-year-old cheetah, ≈4–6 days after it showed clinical signs. At the time of sampling, the remaining 2 cheetahs (animals A and B), which were housed in a separate but conjoining area, had clinically recovered from their respiratory illness. To avoid the additional handling and sedation required for sample collection from the nondomesticated animals, park personnel decided not to collect samples from the recovered cats.

The nasal swab samples were processed according to a standardized procedure distributed by the United States Department of Agriculture National Veterinary Services Laboratory through the National Animal Health Laboratory Network. The protocol used was an approved deviation of the standard operating procedure used for testing swine in the United States. In brief, RNA was recovered by using the MagMAX Viral RNA Isolation Kit (Applied Biosystems, Austin, TX, USA) following the manufacturer’s recommendations. Real-time reverse transcription PCR (qRT-PCR) individually targeting the influenza A matrix (M) gene and the neuraminidase (N1) gene were performed as described (*2*). Positive qRT-PCR test results, with cycle thresholds of 26.83 and 30.23 for the M and N1 genes, respectively, were obtained for animal D. PCR results were confirmed as pandemic (H1N1) 2009 virus by sequence analysis of the PCR amplicon. Animal C had negative test results for M and N1 genes.

A second aliquot of the nasal sample from animal D was inoculated onto trypsin-treated MDCK cells (*3*), and the propagated cytolytic virus was forwarded to St. Jude Children’s Research Hospital for complete genome sequence analysis. The influenza virus isolate was inoculated into ferrets for further characterization of the virus. The United States Department of Agriculture National Veterinary Services Laboratory (Ames, Iowa, USA) was additionally provided an aliquot of the nasal swab specimen. Sequence analyses of the M, N1, and hemagglutinin genes were performed (GenBank accession nos. HMO12479, HMO 12480, and HMO12481) and confirmed the initial diagnostic detection of pandemic (H1N1) 2009 virus.

Whole genome sequencing was performed by using methods recommended by the World Health Organization (www.who.int/csr/resources/publications/swineflu/sequencing_primers/en/). Sequence analysis confirmed the existence of the pandemic (H1N1) 2009 virus (A/Cheetah/California/D0912239/2009, GenBank accession nos. CY092750–CY092757). No unique molecular signatures were detected in the cheetah virus as compared with other pandemic subtype H1N1 viruses isolated from humans. Except for 1 synonymous base-pair mismatch at position 1440 in the hemagglutinin gene, the sequence data matched in the 3 genes sequenced in 2 locations.

To characterize and further assess the pathogenicity profile of the virus, we inoculated 3 groups of 5 ferrets each intranasally with a 10^5.5^ 50% tissue culture infectious dose of A/Cheetah/California/D0912239/2009 or one of the representative strains from humans, A/Tennessee/1–560/09 and A/Ukraine/N6/2009. Animal studies were approved by the St. Jude Animal Care and Use Committee (protocol 428) and were conducted according to applicable laws and guidelines. Ferrets were observed daily, and temperature and weight measurements and nasal washes were obtained for analysis on day 0 and on postexposure days 3, 5, and 7. Weight measurements were performed until day 14.

In all 3 groups, viral shedding was detected on days 3 and 5, with the virus being cleared by day 7. The concentration of shed virus ranged from a 10^3.5^ to a 10^6.3^ tissue culture infectious doses for all 3 tested viruses; differences in the measured virus concentration were not statistically significant (Figure). Slight elevations in body temperature were detected in each group. The bodyweight per day for each ferret was analyzed as the percentage of the original weight; with all 3 viruses, the highest weight loss occurred within the first 7 days after inoculation. Overall, the clinical and virologic course of infection did not differ substantially between infecting viruses (Table).

**Figure:**
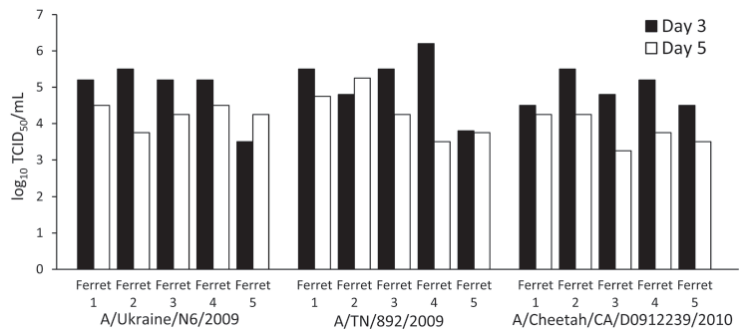
Virus concentration (50% tissue culture infectious dose) in nasal secretions of 3 groups of ferrets (5 animals/group) experimentally infected with different strains of pandemic (H1N1) 2009. In all 3 groups, viral shedding was detected on days 3 and 5, with the virus being cleared by day 7. NW, nasal wash.

**Table:** Clinical and virologic course of infection in 3 groups of ferrets experimentally infected with different strains of pandemic (H1N1) 2009 virus, by strain*

Virus	Average maximum % weight loss (range)	Average maximum increase in temperature, °C	Observed clinical signs
A/Cheetah/CA/D0912239/2010	6.2 (2.3–10.3)	0.9 (0.9–2.7)	Sneezing (2 animals), nasal discharge (1 animal)
A/Ukraine/N6/2009	4.6 (2.8–7.1)	1.2 (0.9, 1.5)	None
A/TN/892/2009	7.0 (3.5–11.0)	1.4 (1.2–1.8)	None

*Five ferrets in each group were intranasally inoculated with a 10^5.5^ 50% tissue culture infectious dose of virus.

## Conclusions

The pandemic (H1N1) 2009 virus was recovered from a captive cheetah showing clinical signs compatible with influenza-like illness. Approximately 7 days before onset of clinical signs in 2 of the 4 affected cheetahs (animals C and D, the animals affected last), it was reported that an animal caregiver, who was not in direct contact with the cheetahs but who had contact with their food and environment, had influenza-like symptoms for 2 days before taking sick leave from work. Attempts to retrospectively confirm the presence of the novel subtype H1N1 virus in this particular worker were not successful, as no specimens were obtained from the worker’s primary care physician at the time or immediately following clinical illness. The 4 cheetahs recovered completely under veterinary care, and a convalescent-phase sample, which tested negative by PCR and virus isolation, was available for only 1 of the 2 earliest affected cheetahs (animal B).

Reverse zoonotic transmission by fomites from contact with an ill animal caregiver is the highly likely scenario for transmission within the cheetah’s restricted environment. Whole-genome sequence analysis showed a single-pair mismatch and 100% amino acid identity between the virus isolated from the cheetah and the pandemic (H1N1) 2009 virus isolated from humans. In addition, the similar data generated from experimentally inoculated ferrets suggest direct transmission of the virus rather than an evolutionary event necessary for species adaptation. The pandemic influenza A (H1N1) virus has been shown to have a high replication rate and expanded tissue tropism pattern that differ from those for seasonal influenza viruses (*4*), which may help explain the observed interspecies transmission of the virus to the cheetah in the reported absence of direct human-animal contact. In the subsequent 2 years, naturally acquired disease has been reported in 10 domestic and wildlife animal species (*5*), including the cheetah reported here.

This case demonstrates the need for a close collaboration between public health and veterinary health agencies in monitoring and understanding the transmission potential of zoonotic infectious agents, including pandemic (H1N1) 2009 virus, that can be transmitted from animals to humans and from humans to animals.
